# RNA Binding Protein CUGBP2/CELF2 Mediates Curcumin-Induced Mitotic Catastrophe of Pancreatic Cancer Cells

**DOI:** 10.1371/journal.pone.0016958

**Published:** 2011-02-11

**Authors:** Dharmalingam Subramaniam, Satish Ramalingam, David C. Linehan, Brian K. Dieckgraefe, Russell G. Postier, Courtney W. Houchen, Roy A. Jensen, Shrikant Anant

**Affiliations:** 1 Department of Molecular and Integrative Physiology, University of Kansas Medical Center, Kansas City, Kansas, United States of America; 2 Department of Surgery, Washington University School of Medicine, St Louis, Missouri, United States of America; 3 Department of Medicine, Washington University School of Medicine, St Louis, Missouri, United States of America; 4 Department of Surgery, University of Oklahoma Health Sciences Center, Oklahoma City, Oklahoma, United States of America; 5 Department of Medicine, University of Oklahoma Health Sciences Center, Oklahoma City, Oklahoma, United States of America; 6 Department of Pathology, University of Kansas Medical Center, Kansas City, Kansas, United States of America; 7 The University of Kansas Cancer Center, University of Kansas Medical Center, Kansas City, Kansas, United States of America; Vanderbilt University Medical Center, United States of America

## Abstract

**Background:**

Curcumin inhibits the growth of pancreatic cancer tumor xenografts in nude mice; however, the mechanism of action is not well understood. It is becoming increasingly clear that RNA binding proteins regulate posttranscriptional gene expression and play a critical role in RNA stability and translation. Here, we have determined that curcumin modulates the expression of RNA binding protein CUGBP2 to inhibit pancreatic cancer growth.

**Methodology/Principal Findings:**

In this study, we show that curcumin treated tumor xenografts have a significant reduction in tumor volume and angiogenesis. Curcumin inhibited the proliferation, while inducing G2-M arrest and apoptosis resulting in mitotic catastrophe of various pancreatic cancer cells. This was further confirmed by increased phosphorylation of checkpoint kinase 2 (Chk2) protein coupled with higher levels of nuclear cyclin B1 and Cdc-2. Curcumin increased the expression of cyclooxygenase-2 (COX-2) and vascular endothelial growth factor (VEGF) mRNA, but protein levels were lower. Furthermore, curcumin increased the expression of RNA binding proteins CUGBP2/CELF2 and TIA-1. CUGBP2 binding to COX-2 and VEGF mRNA was also enhanced, thereby increasing mRNA stability, the half-life changing from 30 min to 8 h. On the other hand, silencer-mediated knockdown of CUGBP2 partially restored the expression of COX-2 and VEGF even with curcumin treatment. COX-2 and VEGF mRNA levels were reduced to control levels, while proteins levels were higher.

**Conclusion/Significance:**

Curcumin inhibits pancreatic tumor growth through mitotic catastrophe by increasing the expression of RNA binding protein CUGBP2, thereby inhibiting the translation of COX-2 and VEGF mRNA. These data suggest that translation inhibition is a novel mechanism of action for curcumin during the therapeutic intervention of pancreatic cancers.

## Introduction

Despite the advances in molecular pathogenesis, pancreatic cancer remains a major unsolved health problem in the United States [1]. Pancreatic cancer is a rapidly invasive, metastatic tumor, which is resistant to standard therapies [2], [3]. At present, single agent based chemotherapy (e.g. Gemcitabine) is the mainstay treatment for metastatic adenocarcinoma of pancreas. Gemcitabine treatment has a tumor response rate of below 10%; similarly none of the available current chemotherapeutic agents have an objective response rate of over 10% [4], [5]. More recently, drug combinations with gemcitabine are also being examined, but it is too early to tell if they are clinically superior. Nevertheless, the magnitude of this problem mandates the need for novel therapeutic agents.

Epidemiological studies suggest that diet plays a major role in the prevention of many cancers. Curcumin, an active ingredient in the spice turmeric, has shown anti-tumor effects in preclinical studies [6], [7]. Anti-tumor properties of curcumin include inhibition of tumor growth and induction of apoptosis [8], [9], [10], [11], [12], [13], [14], [15], [16]. Pilot Phase I clinical trials have shown that curcumin is safe even when consumed at a daily dose of 12 g for 3 months [17], [18], [19]. A most recent phase I/II study demonstrated that combination therapy using 8 g oral curcumin daily with gemcitabine was safe and feasible for patients with pancreatic cancer warranting further investigation into its efficacy [20].

The anti-tumor properties of curcumin have been attributed, at least in part, to its ability to inhibit the expression and activity of cyclooxygenase-2 (COX-2) [21], [22]. It is a rate-limiting enzyme in the arachidonic acid to prostaglandin synthesis pathway. The protein is overexpressed in inflammation and in a variety of cancers. In pancreatic cancers, COX-2 overexpresssion is associated with inhibition of apoptosis, increased tumor invasiveness, and promotion of angiogenesis. The CELF (CUGBP-ETR3-like factors) family of RNA binding proteins is composed of six members that are involved in various cellular processes including mRNA splicing, editing, stability and translation [23]. One of the members, CUGBP2 (also known as CELF2, ETR3, BRUNOL2, Napor2) is expressed ubiquitously, albeit at higher levels in muscle cells [24], [25]. In previous studies, we have demonstrated that when CUGBP2 is overexpressed, the cells undergo mitotic catastrophe [26], [27]. We have also demonstrated that CUGBP2 can bind to AU-rich sequences in the 3′untranslated region of COX-2 mRNA and increase the stability of the mRNA while inhibiting its translation [28]. HuR, a related RNA binding protein with structural similarity to CUGBP2 on the other hand is implicated in increasing COX-2 mRNA stability and enhancing its translation by binding to the same AU-rich sequence elements [29]. A third RNA binding protein that functions as a post-transcriptional regulator of gene expression by recognizing U-rich sequence in the RNA is TIA-1 (T-cell-restricted intracellular antigen-1). TIA-1 has been shown to inhibit mRNA translation in conditions of environmental stress [30], [31]. In this article, we have determined the effect of curcumin on the expression of RNA binding proteins in pancreatic cancer cells.

## Materials and Methods

### Ethics Statement

All animals were handled in strict accordance with good animal practice as defined by the relevant national and/or local animal welfare bodies, and all animal work was approved by the Institutional Animal Care and Use Committee (IACUC) (Protocol#07-009) of University of Oklahoma Health Sciences Center.

### Cells and Reagents

AsPC-1, MiaPaCa-2, Panc-1, BxPC-3 human and Pan02 mouse pancreatic cancer cells (all from American Type Culture Collection, Manassas, VA) were grown in RPMI 1640 containing 10% heat inactivated fetal bovine serum (Sigma Chemical Co, St. Louis, MO) and 1% antibiotic-antimycotic solution (Mediatech Inc, Herndon, VA) at 37°C in a humidified atmosphere of 5% CO_2_. Curcumin was purchased from LKT Laboratories, St Paul, MN.

### Proliferation and Apoptosis assays

To assess proliferation, cells were seeded on to 96 well plates and grown overnight. The cells were then treated with increasing doses of curcumin in RPMI 1640 media containing 10% FBS. Analysis of cell proliferation was performed by enzymatic assay [32]. For apoptosis, caspase 3/7 activity was measured using the Apo-one Homogeneous Caspase-3/7 Assay kit (Promega, Madison, WI).

### Colony formation assay

Briefly, 6 well dishes were seeded with 500 viable cells and allowed to grow for 24 h. The cells were then incubated in the presence or absence of various concentrations of curcumin for up to 48 h. The curcumin-containing medium was then removed, and the cells were washed in PBS and incubated for an additional 10 d in complete medium. Each treatment was done in triplicate. The colonies obtained were washed with PBS and fixed in 10% formalin for 10 min at room temperature and then washed with PBS followed by staining with hematoxylin. The colonies were counted and compared with untreated cells.

### Cell cycle analyses

Cells were treated with curcumin for 12 h and 24 h, and subsequently trypsinized and suspended in phosphate buffered saline (PBS). Single-cell suspensions were fixed using 70% ethanol for 2 h, and subsequently permeabilized with PBS containing 1 mg/ml propidium iodide (Sigma-Aldrich), 0.1% Triton X-100 (Sigma-Aldrich) and 2 µg DNase-free RNase (Sigma-Aldrich) at room temperature. Flow cytometry was done with a FACSCalibur analyzer (Becton Dickinson, Mountain, View, CA), capturing 50,000 events for each sample. Results were analyzed with ModFit LT ™ software (Verity Software House, Topsham, ME).

### Real Time Reverse-Transcription Polymerase Chain Reaction Analysis

Total RNA isolated from MiaPaCa-2, Pan02 cells and tumor xenografts using TRIZOL reagent was reverse transcribed with Superscript II reverse transcriptase in the presence of random hexanucleotide primers (all from Invitrogen, Carlsbad, CA). Complementary DNAs were then used for Real time PCR using Jumpstart Taq DNA polymerase (Sigma Chemical Co, St. Louis, MO) and SYBR Green nucleic acid stain (Molecular Probes, Eugene, OR). Crossing threshold values for individual genes were normalized to β-Actin. Changes in mRNA expression were expressed as fold change relative to control. Primers used in this study were as follows: β-Actin: 5′-GCTGATCCACATCTGCTGG-3′ and 5′-ATCATTGCTCCTCCTCAGCG-3′; COX-2: 5′-GAATCATTCACCAGGCAAATTG-3′ and 5′-TCTGTACTGCGGGTGGAACA-3′; VEGF: 5′-AGCGCAAGAAATCCCGGTA-3′ and 5′-TGCTTTCTCCGCTCTGAGC-3′; HuR: 5′-GTGAACTACGTGACCGCGAA-3′and 5′-GACTGGAGCCTCAAGCCG-3′; CUGBP2: 5′-TGCTTCAACCCCCAACTCC-3′ and 5′-GTCCTTGCAGAGTCCCGAGA-3′; TIA-1: 5′-ACCCATCTGTTGAATGGCGT-3′and 5′- GGCAACAGGAAAGTCTAAGGGAT-3′; Cyclin D1: 5′-AATGACCCCGCACGATTTC-3′ and 5′-TCAGGTTCAGGCCTTGCAC-3′.

### RNA Stability assay

Cells were treated with curcumin for 2 h. Actinomycin-D (10 µg/ml final concentration), a potent inhibitor of mRNA synthesis was added to the cells and total mRNA was extracted at 0–8 h. RNA was subjected to Real time PCR as described above. Data is presented as relative to control cells, at the time of addition of actinomycin D.

### Western Blot Analysis

Cell lysates were subjected to polyacrylamide gel electrophoresis and blotted onto Immobilin polyvinylidene difluoride membranes (Millipore, Bedford, MA). Antibodies were purchased from Cell Signaling Technology (Beverly, MA), Abcam Inc (Cambridge, MA) and Santa Cruz Biotechnology Inc (Santa Cruz, CA) and specific proteins were detected by the enhanced chemiluminescence system (Amersham Pharmacia Biotech, Piscataway, NJ).

### Immunoprecipitation

For immunoprecipitation, the cells were fixed with 1% formalin. Lysates were prepared and immunoprecipitated with anti-CUGBP2 antibody. The pellet and supernatant were subsequently incubated at 70°C for 1 h to reverse the cross-links, and RNA was purified and subjected to RT-PCR to determine COX-2 and VEGF mRNA levels.

### siRNA

Sequence targeting the CUGBP2 mRNA 5′-GCAAACCUUACUGAUCCUA-3′ (Accession mumber ns. NM_001025076) and a scrambled control siRNA not matching any of the human genes were obtained from Ambion Inc, Texas, USA and transfected with siPORT transfection reagent (Ambion).

### Pan02 xenograft tumors

Five-week-old male athymic nude mice purchased from Jackson Laboratories were utilized for *in vivo* experiments. The mice were maintained with water and standard mouse chow *ad libidum* that is used in protocols approved by the University's Animal Studies Committee. Animals were injected with 1×10^6^ Pan02 cells in the left and right flank and allowed to form xenografts. One week following planting the cells and after observing the presence of a palpable tumor, curcumin (200 µg/kg body weight) in 5% Na_2_ HCO_3_ buffer alone was administered intraperitoneally daily for 23 d. Tumor size was measured weekly. At the end of treatment the animals were sacrificed, and the tumors were removed, weighed, and prepared for use in histology (hematoxylin & eosin, COX-2, VEGF, and CD31) and gene expression studies.

### Immunocytochemistry and immunohistochemistry

Cells were plated on to coverslip and allowed to grow for 24 h. The cells were then fixed with 10% buffered formalin for 10 min and subsequently washed with PBS. The cells were then permeabilized with PBS containing 0.5% Triton X-100 for 10 min. The coverslips were incubated with rabbit anti-cyclin B1 (Santa Cruz Biotechnologies, Santa Cruz, CA) and rabbit anti-Cdc2 antibodies (Abcam Inc, Cambridge, MA), followed by biotinylated anti-rabbit IgG. The slides were further processed using Vectastatin ABC kit (Vector Laboratories, Burlingame, CA) followed by DAB staining.

Tissues embedded in paraffin were cut to a section of 4 µM, deparaffinized and treated with citrate buffer. Then they were blocked with Avidin/Biotin for 20 min. The slides were incubated with anti-COX-2 or VEGF or CD31 or HuR or CUGBP2 or TIA-1 or Cyclin D1 for overnight at 4°C. Next the slides were treated with the secondary antibody such as HRP- goat anti-rabbit for subsequent COX-2, VEGF, HuR, CUGBP2, TIA-1, and Cyclin D1 staining. For CD31staining goat anti-rat HRP was utilized. Each secondary antibody was incubated for one hour and then developed with DAB (Sigma Aldrich). Finally, the slides were counterstained with hematoxylin.

### Statistical analysis

All values are expressed as the mean ± SEM. Data was analyzed using a unpaired 2-tailed t test. A *P* value of less than 0.05 was considered statistically significant.

## Results

### Curcumin inhibits tumor growth of Pan02 tumor xenografts and angiogenesis

**Figure 1:**
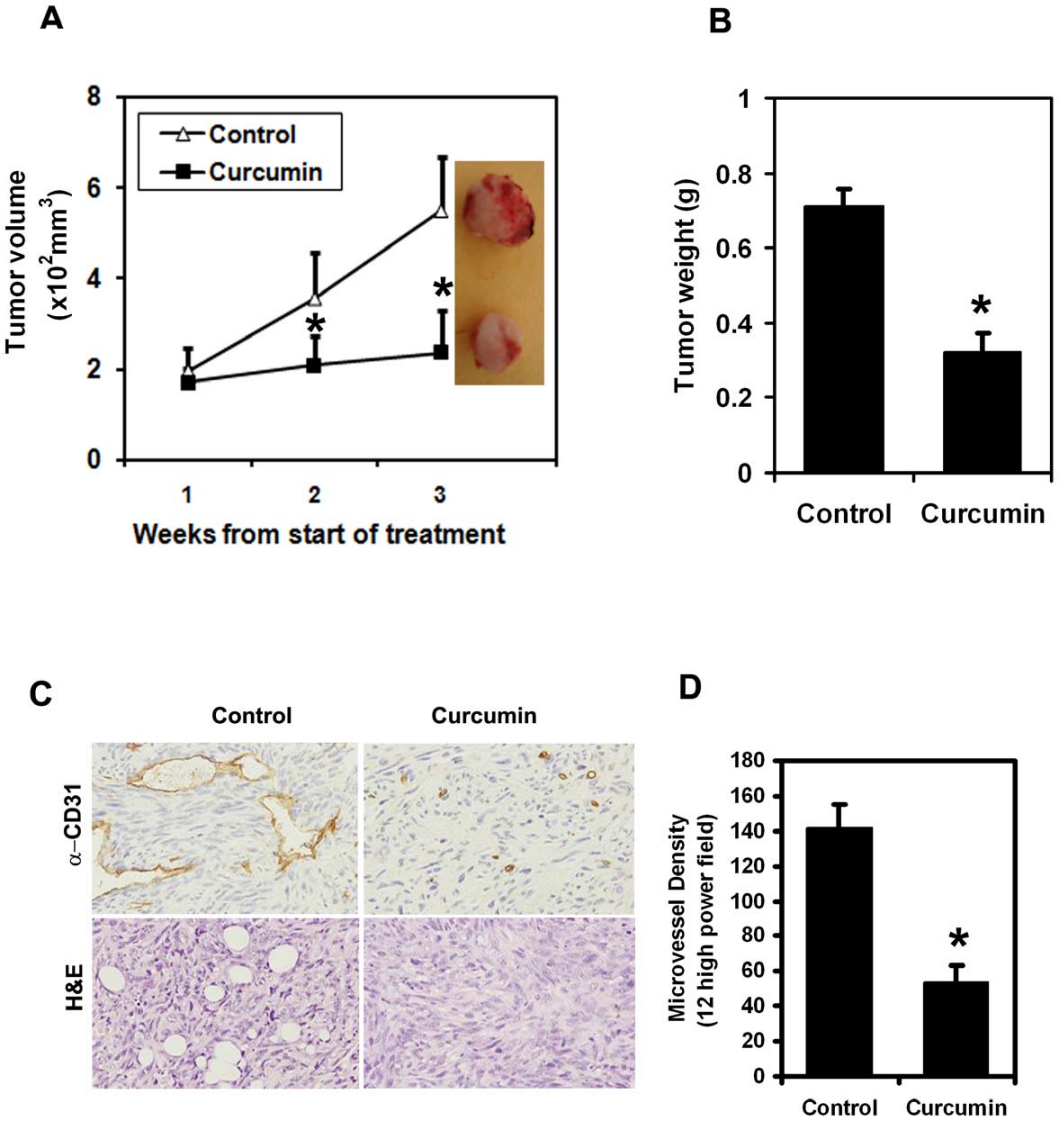
Curcumin inhibits growth of Pan02 tumor xenografts. (A) Nude mice carrying Pan02 cell tumor xenografts in the flanks were administered curcumin intraperitoneally for 3 weeks. There was a significant reduction in tumor size from curcumin-treated animals when compared to untreated controls (*p<0.05). (B) Curcumin treatment resulted in significantly lower tumor weight when compared to controls (*p<0.05). (C) Tumor sections were stained for CD31, an endothelial cell specific surface marker and with hematoxylin and eosin (H&E). A representative figure is presented showing significant reduction in microvessels. (D) The number of microvessels in the tumor tissues were counted in 12 high power fields and averaged. Data shows that microvessel density was significantly reduced in the xenografts of curcumin treated animals (*p<0.05).

To begin evaluating the role of curcumin on tumor proliferation *in vivo*, we examined the ability of curcumin to suppress the growth of mouse pancreatic cancer cell xenografts in nude mice. Pancreatic cancer cell induced xenograft tumors were allowed to develop and grow to a size of 200 mm^3^ following which curcumin was administered intraperitoneally for three weeks daily. Curcumin significantly inhibited the growth of the tumor xenografts (Fig. 1A). The excised tumors from control animals ranged from 700 to 800 mg while those treated with curcumin weighed less than 400 mg (Fig. 1B). In addition, tumor volume was significantly decreased (Fig. 1A). There was no apparent change in liver weight, spleen weight, or body weight in the animals (data not shown). These data imply that curcumin is a potent therapeutic agent to treat pancreatic cancers and is relatively non-toxic to the mice. We also determined the effect of curcumin on tumor vascularization by staining for the endothelial specific antigen CD31. As shown in Figure 1C, curcumin treatment significantly lowered CD31 staining and obliterated the tumor vessels suggesting decreased angiogenesis. We also calculated the microvessel density and found it to be significantly decreased following curcumin treatment (Fig. 1D).

### Curcumin inhibits pancreatic cancer cell growth

**Figure 2:**
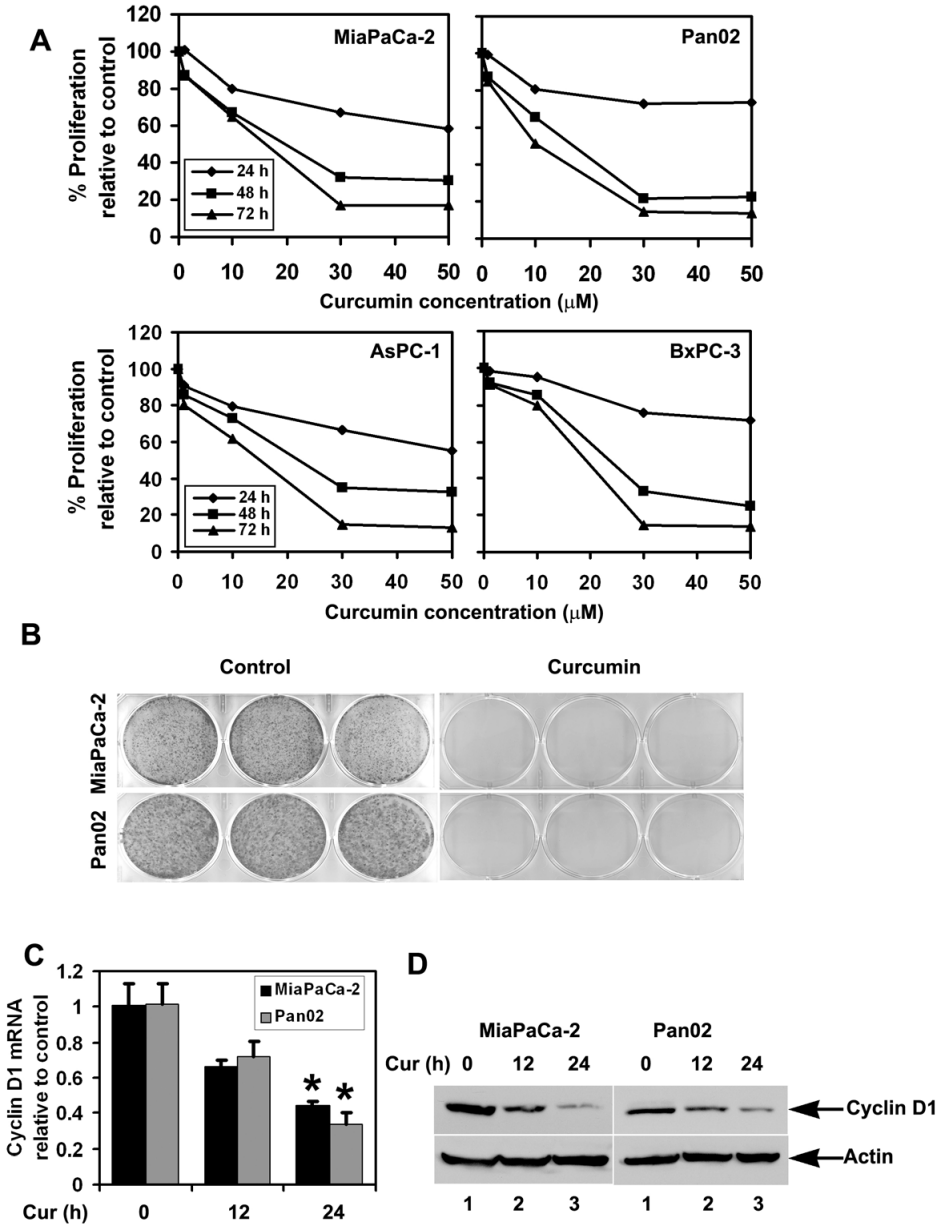
Curcumin inhibits pancreatic cancer cell growth. (A) Curcumin inhibits proliferation of pancreatic cancer cells. MiaPaCa-2, Pan02, AsPC-1 and BxPC-3 cells were incubated with curcumin (1–50 µM) for up to 72 h. Cell proliferation was analyzed using hexosaminidase enzyme activity. Curcumin treatment resulted in a significant dose-and time-dependent decrease in cell proliferation in all the cells when compared with untreated controls. (B) Curcumin inhibits colony formation. MiaPaCa-2 and Pan02 cells were incubated with 30 µM of curcumin for 24 h and colonies were allowed to form by incubating in regular media containing 10% FBS for an additional 10 d. Treatment with curcumin inhibited colony formation. A representative of three independent experiments is shown. (C) Expression of cyclin D1 is suppressed at 24 h. RNA from MiaPaCa-2 and Pan02 cells incubated with 30 µM curcumin were subjected to Real time PCR for cyclin D1 mRNA expression. There was significant suppression of the mRNA at 24 h but not at 12 h (*p<0.05). (D) Lysates from MiaPaCa-2 or Pan02 incubated with 30 µM curcumin were analyzed by western blotting for cyclin D1 expression levels. Curcumin treatment inhibits cyclin D1 mRNA and protein expression.

To determine the mechanism of curcumin-mediated growth arrest, we studied pancreatic cancer cells *in vitro*. For this we used five different cultured cell lines MiaPaCa-2, Panc-1, AsPC-1, BxPC-3 and Pan02. Curcumin significantly suppressed the proliferation of pancreatic cancer cell lines MiaPaCa-2, Pan02, AsPC-1, BxPC-3 and Panc-1 in a dose and time dependent manner. This anti-proliferation effect on tumor cells was seen within a 24 h period, which continued to significantly increase over the next 72 h (Fig. 2A). To determine the long-term effect of curcumin treatment, cells were treated with 30µM curcumin for 24 h, following which the cells were allowed to grow in normal media. Curcumin treatment suppressed colony formation in all pancreatic cancer cell lines (Fig. 2B), suggesting that curcumin's effects on the tumor cells was irreversible. Cyclin D1 is a cell cycle regulatory protein that regulates the G1 to S-phase transition of the cell cycle and functions as a cofactor for several transcription factors [33]. Cyclin D1 overexpression has been linked to the development and progression of cancer [34]. In both MiaPaCa-2 and Pan02 cells, curcumin treatment resulted in reduced cyclin D1 expression at 24 h (Fig. 2C, D). Furthermore, cyclin A2, which regulates S/G2 progression, was downregulated potentially slowing progression of cells out of S phase (data not shown).

### Curcumin induces G2-M cell cycle arrest before mitotic catastrophe

**Figure 3:**
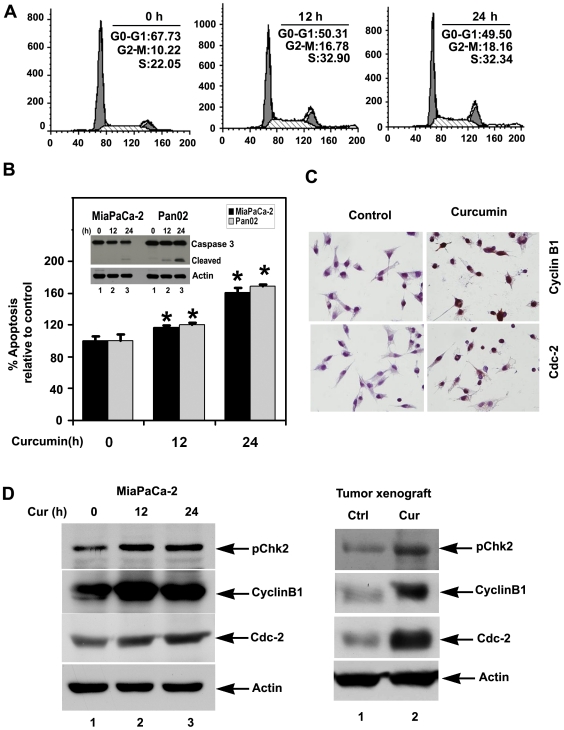
Curcumin induces mitotic catastrophe. A) Cell cycle profiles of MiaPaCa-2 cells treated with curcumin for 12 h and 24 h and were determined by flow cytometry using propidium iodide staining for DNA content. Curcumin treatment significantly increased cells in the S and G_2_M phase of cell cycle within 12 h. (B) Curcumin treatment induces apoptosis. MiaPaCa-2 and Pan02 cells incubated with 30 µM of curcumin were analyzed for apoptosis by caspase 3/7 activation. Curcumin treatment increased the number of cells undergoing apoptosis compared to untreated controls (*p<0.05). (Inset) Curcumin induces caspase 3, an apoptosis mediator. Lysates from MiaPaCa-2 or Pan02 cells incubated with 30µM curcumin were analyzed by western blotting for caspase 3 protein. Curcumin treated cells shows cleaved (activated) caspase 3 while untreated cells have no cleaved caspase-3. (C) Curcumin treatment increased the levels of cyclin B1, Cdc-2 and phosphorylated checkpoint kinase Chk2. Lysates from curcumin treated MiaPaCa-2 cells (left) and Pan02 tumor xenografts (right) were analyzed by western blotting for phospho Chk2, and cyclin B1 and Cdc-2 protein expression levels. Curcumin treatment phosphorylates checkpoint kinases and increased levels of cyclin B1 and Cdc-2 in both MiaPaCa-2 cells and Pan02 tumor xenografts. (D) Curcumin treatment results in nuclear localization of cyclin B1 and Cdc-2. Immunocytochemistry of curcumin treated with MiaPaCa-2 cells both the cyclinB1 and Cdc-2 protein levels were predominately in the nucleus compared than untreated cells. Phosphorylation of Chk2, coupled with increased expression and nuclear translocation of cyclin B1 and Cdc-2 demonstrates mitotic progression of cells.

We next determined whether curcumin affects cell cycle progression and the consequence of this effect. Curcumin induced growth arrest of the MiaPaCa-2 within 24 h at the G_2_M stage (Fig. 3A). Similar results were observed in Panc-1 and Pan02 cells (data not shown). Curcumin is known to induce apoptosis in variety of cancer cells. Caspase-3 and 7 are key effector molecules known to induce apoptosis in variety of cancer cells by amplifying the signal from initiator caspases, such as caspase 8 or caspase 10 [35], [36]. Capsase 3 and 7 were increased within 24 h in the MiaPaCa-2 cells line treated with curcumin suggesting the induction of apoptosis (Fig. 3B). Western blot analyses of MiaPaCa-2 and Pan02 cell lysates demonstrated a significant increase in the activated caspase-3 in curcumin treated cells (Fig. 3B inset). These data suggest that curcumin treated tumor cells were undergoing apoptosis. To determine whether an arrest at the G2 phase or lack of progression to mitosis is contributing to the higher level of cells seen in G_2_M phase, we assessed the expression, and localization of cyclin B1 and the serine/thereonine kinase Cdc-2. Cyclin B1/Cdc-2 complex plays a significant role in the G_2_M phase transition of cell cycle. To induce mitosis, cyclin B1 and Cdc-2 heterodimerize and localize to the nucleus, which in turn activates the enzymes that regulates chromatin condensation, nuclear membrane breakdown and mitosis specific microtubule reorganization [37], [27]. Immunocytochemical analysis demonstrated significantly higher nuclear levels of both cyclin B1 and Cdc-2 in curcumin treated cells suggesting that curcumin treated cells were undergoing mitotic catastrophe (Fig. 3C). In addition to this, western blot analysis demonstrated increased expression of both cyclin B1 and Cdc-2 in curcumin treated cells and phosphorylation of Chk2 kinase in both MiaPaCa-2 cells in culture and the Pan02 tumor xenograft tissues (Fig. 3D). Given that the cells were undergoing apoptosis at the same time that they were in G2-M arrest, suggests that curcumin induces the cells to undergo mitotic catastrophe.

### Curcumin inhibits COX-2 and VEGF expression while inducing CUGBP2 and TIA-1

**Figure 4:**
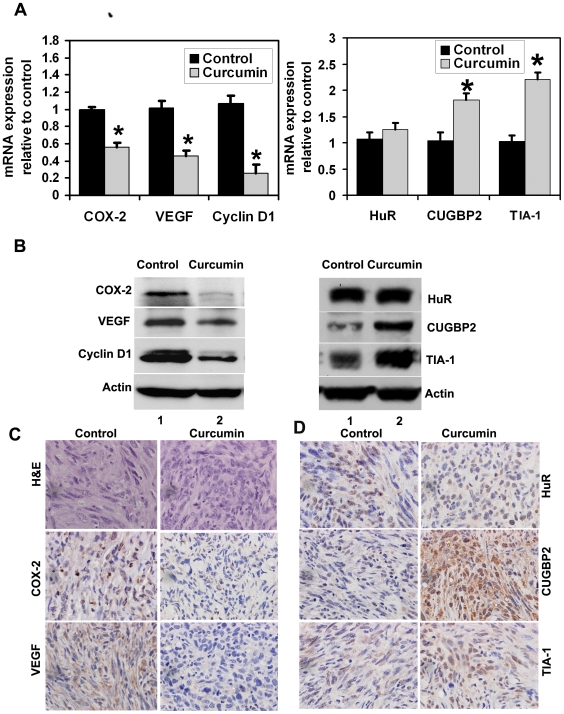
Curcumin inhibits COX-2, VEGF and Cyclin D1 expression in the tumors while inducing CUGBP2 and TIA-1. (A) Total RNA from Pan02 tumor xenografts were subjected to Real time PCR. Curcumin treatment resulted in reduced COX-2, VEGF and cyclin D1 mRNA levels when compared to controls. On the other hand, CUGBP2 and TIA-1 mRNA expression was increased (*p<0.05). Data is an average from xenografts in 5 mice. (B) Western blot analysis demonstrated that tissue lysates from the curcumin treated animals have significantly lower levels of COX-2, VEGF, and cyclin D1 proteins but increased levels of CUGBP2 and TIA-1 proteins. (C) Immunohistochemistry demonstrates that curcumin treatment significantly reduced the expression of COX-2 and VEGF. (D) Immunohistochemistry demonstrates increased expression of CUGBP2 and TIA-1 in the tumor xenografts.

COX-2 plays a significant role in carcinogenesis, including increased invasiveness, promotion of angiogenesis and resistance to apoptosis [38]. We therefore determined its expression in tumor xenografts. Real time PCR analyses demonstrated that COX-2 mRNA expression was significantly lower in curcumin treated tumor xenografts when compared to the control tumors (Fig. 4A). This was further confirmed by western blot and immunohistochemistry analyses, where lower levels of COX-2 protein were observed in the curcumin-treated tissues (Fig. 4B,C). Prostaglandins and the other tumor promoters are known to induce the expression of VEGF in epithelial cells which promotes angiogenesis and thereby tumor growth [39]. VEGF mRNA and protein expression was analyzed and was also found to be significantly lower in curcumin treated tumor xenografts when compared to the control tumors (Fig. 4A,B). Immunohistochemistry also demonstrated that curcumin treatment significantly reduced the VEGF staining when compared to control tumor (Fig. 4C). Cyclin D1 is a cell cycle regulatory protein that regulates the G1 to S-phase transition and functions as a cofactor for several transcription factors [40]. Cyclin D1 overexpression has also been linked to the development and progression of cancer [34]. Curcumin treatment inhibited cyclin D1 expression in the tumor xenografts suggesting that it inhibits cancer cell proliferation (Fig. 4A,B). A number of RNA-binding proteins have been identified to recognize ARE-containing sequences and regulate mRNA stability. Members of the CELF (CUGBP2) and ELAV (HuR) family of RNA binding proteins are implicated in various cellular processes including mRNA splicing, editing, stability and translation [23], [41]. We have previously demonstrated that CUGBP2 is induced in cells undergoing apoptosis and functions to inhibit COX-2 mRNA translation [28], [42]. T-cell- restricted intracellular antigen-1 (TIA-1) is also an RNA binding protein that functions as a translational suppressor, especially under conditions of stress [43]. Here we observed that curcumin treatment resulted in a significant increase in both CUGBP2 and TIA-1 mRNA and protein expression in the tumor xenografts (Fig. 4A, B, D). On the other hand, there was no significant change observed in both HuR mRNA and protein levels in response to curcumin (Fig. 3A, B, D).

### Curcumin inhibits COX-2 and VEGF mRNA translation in pancreatic cancer cells

**Figure 5:**
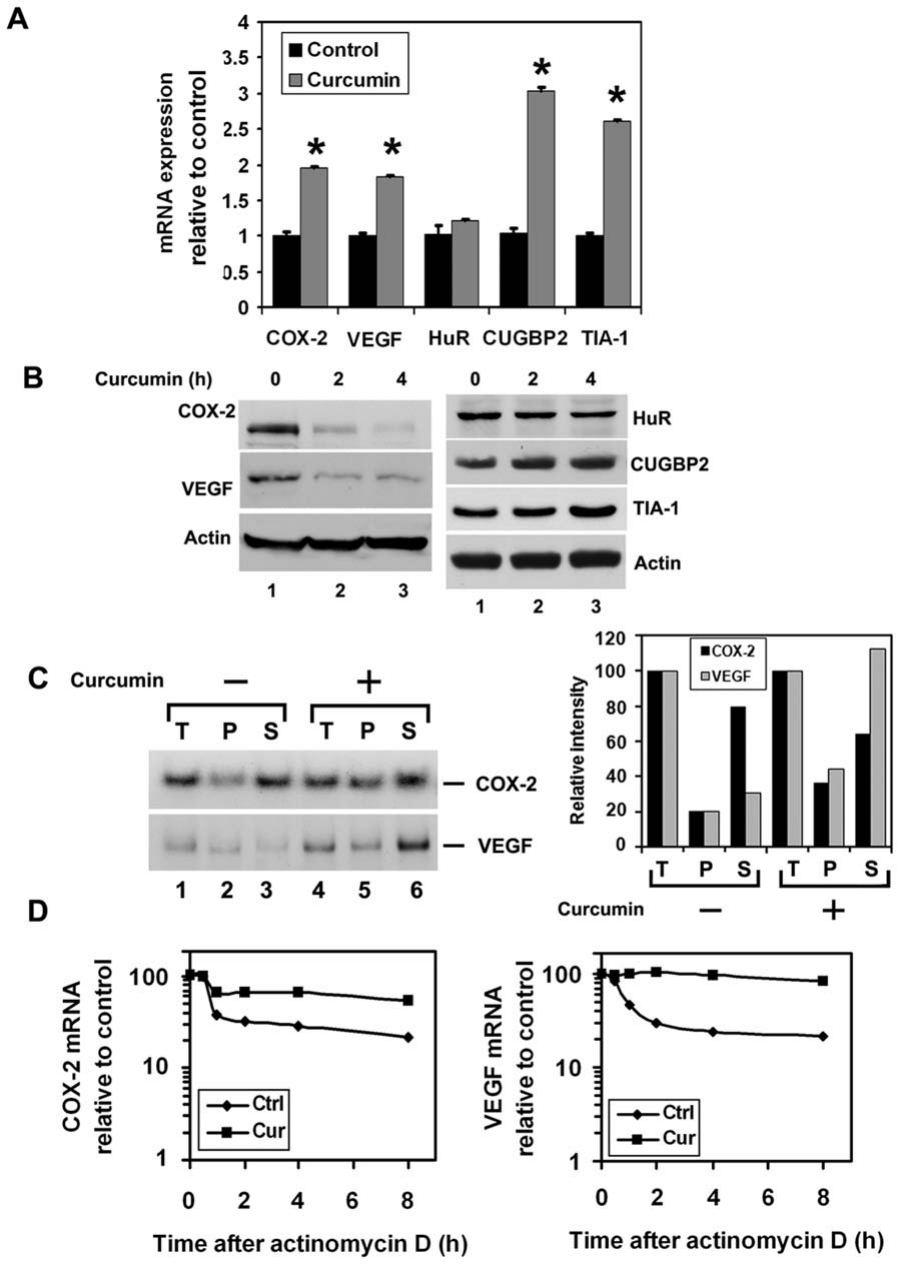
Curcumin inhibits COX-2 and VEGF protein expression in pancreatic cancer cells while inducing CUGBP2 and TIA-1. (A) mRNA expression. MiaPaCa-2 cells were treated with curcumin for 2 h. Curcumin treatment increased the levels of COX-2, VEGF, CUGBP2 and TIA-1 mRNA. There was no significant change of HuR mRNA expression (* p<0.05). Data from three independent experiments. (B) Western blot analyses demonstrate that lysates with curcumin treated MiaPaCa-2 cells have lower levels of COX-2 and VEGF proteins and increasing levels of CUGBP2 and TIA-1. Representative of three independent experiments. (C) (Left panel), Increased binding of CUGBP2 binding to COX-2 or VEGF mRNA following curcumin treatment. Whole cell extract (T) from curcumin-treated cells were immunoprecipitated with anti-CUGBP2 antibody, and RNA from the immunoprecipitates (P) and supernatant (S) were isolated and subjected to RT-PCR for COX-2 and VEGF mRNA. Data demonstrates increased COX-2 or VEGF mRNA in the pellet of curcumin-treated cells. (Right panel) Data was quantified and there was a clear increase in COX-2 and VEGF mRNA in the pellet of curcumin treated cells. (D) MiaPaCa-2 cells were treated with curcumin for 2 h and the stability of COX-2 and VEGF mRNA were determined following addition of actinomycin D (final concentration: 10 µg/ml). Curcumin increases the half-life of COX-2 mRNA from 30 min to 8 h. Similarly, curcumin increased the half-life of VEGF mRNA from 30 min to 8 h. Data demonstrates curcumin treatment significantly increased stability of COX-2 or VEGF mRNA.

Previous studies have demonstrated increased levels of COX-2 mRNA and protein expression in pancreatic adenocarcinomas [38]. Therefore, we next determined the effects of curcumin treatment on COX-2 expression. Curcumin treatment significantly increased COX-2 mRNA levels in MiaPaCa-2 cells (Fig. 5A). However, there was a significant reduction in COX-2 protein levels (Fig. 5B). Similar results were observed on Pan02 cells (data not shown). We also determined the effect of curcumin on VEGF gene expression in the cells. Curcumin treatment significantly increased VEGF mRNA while inhibiting protein levels in MiaPaCa-2 cells (Fig. 5A,B). Similar results were observed in Pan02 cells (data not shown). On the other hand, curcumin significantly increased both CUGBP2 and TIA-1 mRNA and protein expression (Fig. 5A,B). However, there was no significant change observed with HuR expression (Fig. 5A,B). In previous studies, we have demonstrated that CUGBP2 binds to COX-2 3′UTR to inhibit its translation [28]. Given that COX-2 mRNA levels are higher, but protein levels are lower in response to curcumin treatment, we determined whether curcumin increases the binding of CUGBP2 to COX-2 mRNA. Immunoprecipitation coupled RT-PCR demonstrated that there was a higher level of CUGBP2 binding to COX-2 and VEGF mRNA when compared to control, untreated cells (Fig. 5C). Furthermore, actinomycin D experiments demonstrated that curcumin treatment resulted in significantly higher levels of COX-2 mRNA stability, the half-life increasing from 30 min in the untreated cells to ∼8 h (Fig. 5D). Similar results were observed with VEGF; the half-life of VEGF mRNA also increased from 30 min to >8 h in curcumin treated cells (Fig. 5D).

### Curcumin mediated COX-2 and VEGF mRNA stability requires CUGBP2

**Figure 6:**
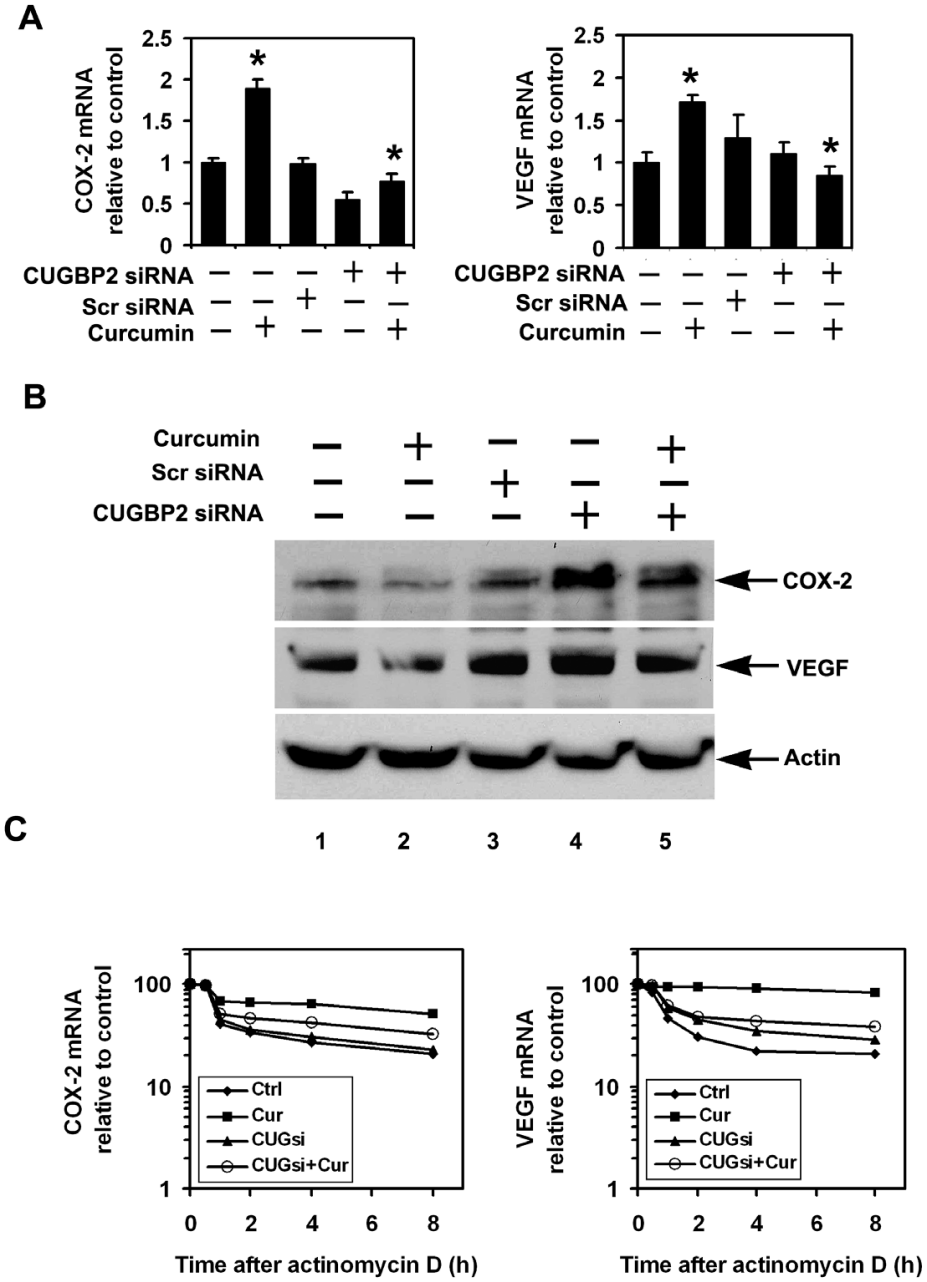
Curcumin mediated COX-2 and VEGF mRNA stability requires CUGBP2. (A) CUGBP2 is necessary for curcumin-induced COX-2 and VEGF mRNA levels. MiaPaCa-2 cells were transfected with CUGBP2 siRNA for 72 h and subsequently treated with curcumin for 2 h. Knockdown of CUGBP2 significantly decreased curcumin-mediated increase in COX-2 and VEGF mRNA expression (*p<0.05). Data from three independent experiments. (B) Western blot analyses of MiaPaCa-2 cell lysates demonstrate that knockdown of CUGBP2 partially restored the expression of COX-2 and VEGF proteins. (C) Knockdown of CUGBP2 reduces curcumin-induced stability of COX-2 and VEGF mRNA. MiaPaCa-2 cells were treated with curcumin for 2 h and the stability of COX-2 and VEGF mRNA were determined following addition of actinomycin D (10 µg/ml). Curcumin increases the half life of COX-2 and VEGF mRNA. However, knockdown of CUGBP2 using specific siRNA before curcumin treatment decreased the stability of both COX-2 and VEGF mRNA.

We have previously shown that CUGBP2 binding to COX-2 3′UTR increases the stability of COX-2 mRNA, while inhibiting its translation [28]. We therefore determined the effect of CUGBP2 knockdown on curcumin-mediated translation suppression of COX-2 and VEGF mRNA. Real Time PCR measurements demonstrated that curcumin treatment resulted in significantly higher levels of COX-2 and VEGF mRNA, which was reduced to control levels when CUGBP2 expression was knockdown (Fig. 6A). We next determined if there were any effects on protein expression. Curcumin treatment significantly decreased the levels of both COX-2 and VEGF protein. However, when CUGBP2 was knocked down with a specific siRNA, COX-2 and VEGF proteins levels were not affected by curcumin treatment (Fig. 6B). We also determined whether curcumin-mediated effects on COX-2 and VEGF mRNA stability are affected by CUGBP2 knockdown. While curcumin treatment increased the stability of COX-2 and VEGF mRNA, there was a lesser effect when CUGBP2 was also knocked down using specific siRNA. The half-life of COX-2 mRNA was increased from 30 min to ∼8 h following curcumin treatment, which was reduced to ∼2 h when CUGBP2 expression was inhibited (Fig. 6C). Similar results were obtained for VEGF where knockdown of CUGBP2 reduced the curcumin-mediated stability from 8 h to 1 h (Fig. 6C). These data suggest that the curcumin mediated increase in COX-2 and VEGF mRNA stability occurs in part through CUGBP2.

## Discussion

Pancreatic cancer is one of the most lethal cancers and has emerged as one of the leading causes of cancer-related deaths in the western world, with most patients dying of their disease with in one year of diagnosis. The significant morbidity, toxicity and poor response rates of current chemotherapy regimens have led to searches for less toxic alternative therapies. Previous studies have shown that curcumin suppresses the proliferation of a variety of tumor cells, including pancreatic cancer [11], [12], [44], [45]. Although earlier studies have attributed the anti-tumor properties of curcumin to the inhibition of COX-2 and VEGF expression, the mechanism by which this occurs is not completely understood. RNA binding protein HuR has been demonstrated to bind to the COX-2 3′UTR and enhance the stability and translation of COX-2 mRNA [46]. In this case, we have demonstrated CUGBP2 binds to the same sequence, but inhibits COX-2 mRNA translation [28]. Moreover, we have demonstrated that CUGBP2 induces cells to undergo mitotic catastrophe. This intrigued and inspired us to further investigate the role of these RNA binding proteins in curcumin mediated pancreatic tumor growth inhibition.

Our *in vivo* xenograft studies demonstrated that curcumin treatment resulted in the downregulation of COX-2 and VEGF proteins coupled with a reduction in microvessel density. In the studies conducted with pancreatic cancer cells in culture, 2 h following curcumin treatment there was an increase in the levels of tumor promoting genes COX-2 and VEGF mRNA. However, there was a significant reduction in the levels of both proteins suggesting translation inhibition. A number of RNA binding proteins have been identified that regulate the stability and translation of AU-rich containing mRNAs. Here, we show that curcumin increased the expression of two RNA binding proteins CUGBP2 and TIA-1. Both proteins have been demonstrated to inhibit translation of target mRNAs including COX-2 and VEGF upon binding to the AU-rich sequences in the 3′untranslated region. Moreover, TIA-1 has been shown to bind untranslated mRNAs and localize them into discrete cytoplasmic foci called stress granules (SGs) [47], [48]. These foci have been hypothesized to function as dynamic holding sites of mRNA and molecular decisions are made on their subsequent engagement with the translation or degradation machineries.

Previous studies have demonstrated that curcumin inhibits the expression of COX-2 and VEGF [21], [22]. Here, we performed a time course for COX-2 and VEGF mRNA levels from 2 h to 24 h after curcumin treatment. At 2h, COX-2 and VEGF mRNA levels increase up to 2-fold but decrease after 4h. We believe the cells initially upregulate COX-2 and VEGF mRNA to counter the effects of curcumin, but then CUGBP2 is also upregulated to inhibit their translation.

We demonstrate that curcumin effectively inhibits the growth of pancreatic tumor xenografts in part through the induction of mitotic catastrophe. As such, mitotic catastrophe is a poorly defined type of cell death linked to the abnormal activation of cyclin B/Cdk1. This can occur at various steps during the G_2_M phase of cell cycle. One form could occur with a significant delay in the G2 checkpoint, while another occurs around metaphase in a partially p53-dependent fashion. In this case, the cells die due to caspase activation and mitochondrial membrane depolarization. Prevention of caspase activation in this setting is believed to induce cytogenic abnormalities thereby leading to oncogenesis. Our data, demonstrating that curcumin induces mitotic catastrophe in part through induction of CUGBP2 expression is consistent with this observation. In previous studies, we have demonstrated that CUGBP2 induces cancer cells to undergo mitotic catastrophe when overexpressed. Further studies are necessary to determine what percentage of pancreatic cancer cells can overcome curcumin-induced apoptosis in the setting of CUGBP2 downregulation.

Our *in vitro* study results show that curcumin can effectively suppress cell proliferation within 24 h. Moreover, the inhibitory effects on tumor cells appears to be sustained and irreversible after 24 h of treatment. Our data with cyclin D1 is intriguing in that there was a reduction in its expression at 24 h. This should have resulted in cells undergoing G0–G1 arrest since the protein is responsible for progression through the G0–G1/S phase transition [40]. However, we believe this reduction is because the cells have undergone G_2_M arrest at 24 h following curcumin and are not actively progressing through the cell cycle. In addition, curcumin downregulated the expression of cyclin A2, a protein that regulates S-G_2_ progression, potentially slowing progression of cells out of S phase. Further studies are required to determine if this is compensatory mechanism from the cancer cells to protect from mitotic catastrophe. Taken together, these data suggest that curcumin can affect various stages of cell cycle progression of pancreatic cancer cells, but the significant effect is the induction of apoptosis during mitosis leading to mitotic catastrophe.

In conclusion, these studies provide mechanistic evidence that curcumin mediated inhibition of COX-2 and VEGF expression occurs through increased expression of RNA binding proteins CUGBP2 and TIA-1 resulting in mitotic catastrophe of the tumor cells. Considering that curcumin treated cells were actively transiting through mitosis before undergoing mitotic catastrophe, it further implies that inducing CUGBP2 and TIA-1 might be an effective chemotherapeutic strategy to treat pancreatic cancer cells. Moreover, the possibility exists that induction of these RNA binding proteins might enhance the sensitivity to chemotherapeutic agents, thereby reducing the bystander toxic effects of the compounds on neighboring cells.
